# Toll-Like Receptor 4 in Bone Marrow-Derived Cells Contributes to the Progression of Diabetic Retinopathy

**DOI:** 10.1155/2014/858763

**Published:** 2014-08-17

**Authors:** Hui Wang, Haojun Shi, Jing Zhang, Guoliang Wang, Jinxiang Zhang, Fagang Jiang, Qing Xiao

**Affiliations:** ^1^The Department of Medical Genetics, Tongji Medical College Affiliated with Huazhong University of Science and Technology, 13 Hangkong Road, Wuhan, Hubei 430022, China; ^2^Radiology Department, Union Hospital Affiliated with Huazhong University of Science and Technology, 1277 Jiefang Avenue, Wuhan, Hubei 430022, China; ^3^The Department of Ophthalmology, Union Hospital Affiliated with Huazhong University of Science and Technology, 1277 Jiefang Avenue, Wuhan, Hubei 430022, China; ^4^The Department of Ophthalmology, No. 1 Hospital of Xian City, 30 Powder Lane, South Street, Xi'an, Shanxi 710002, China; ^5^Department of Surgery, University of Pittsburgh Medical Center, NW607 MUH, 3459 Fifth Avenue, Pittsburgh, PA 25213, USA; ^6^The Department of Emergency Surgery, Union Hospital Affiliated with Huazhong University of Science and Technology, 1277 Jiefang Avenue, Wuhan, Hubei 430022, China

## Abstract

Diabetic retinopathy (DR) is a major microvascular complication in diabetics, and its mechanism is not fully understood. Toll-like receptor 4 (TLR4) plays a pivotal role in the maintenance of the inflammatory state during DR, and the deletion of TLR4 eventually alleviates the diabetic inflammatory state. To further elucidate the mechanism of DR, we used bone marrow transplantation to establish reciprocal chimeric animals of TLR4 mutant mice and TLR4 WT mice combined with diabetes mellitus (DM) induction by streptozotocin (STZ) treatment to identify the role of TLR4 in different cell types in the development of the proinflammatory state during DR. TLR4 mutation did not block the occurrence of high blood glucose after STZ injection compared with WT mice but did alleviate the progression of DR and alter the expression of the small vessel proliferation-related genes, vascular endothelial growth factor (VEGF), and hypoxia inducible factor-1*α* (HIF-1*α*). Grafting bone marrow-derived cells from TLR4 WT mice into TLR4 mutant mice increased the levels of TNF-*α*, IL-1*β*, and MIP-2 and increased the damage to the retina. Similarly, VEGF and HIF-1*α* expression were restored by the bone marrow transplantation. These findings identify an essential role for TLR4 in bone marrow-derived cells contributing to the progression of DR.

## 1. Introduction

Diabetic retinopathy (DR) is one of the most common microangiopathic complications of patients with diabetes mellitus (DM) [1]. DR is an ocular manifestation of DM, affecting up to 80% of all patients who have had DM for 10 years or more.

The mechanism of DR is far from fully understood. Several studies have suggested that low-grade inflammation is an important characteristic of DR [2]. Its core pathological process is elicited by inflammatory injury to the vascular endothelial cells in the retina, which is followed by the angiogenesis of small vessels in the early stage [3–5].

Toll-like receptors (TLRs) belong to a family of transmembrane receptors involved in damage-associated molecular pattern-induced immune activation. One of the most documented types of TLR is toll-like receptor 4 (TLR4), which is expressed on various cell types [6, 7]. Previous studies implicated TLR4 in the regulation of a variety of inflammatory or immune-related disorders [8–11].

Recent findings have demonstrated that TLR4 polymorphisms are associated with the inflammatory state during DM and with complications including DR [12–15]. TLR4 has been shown to play an important role in DR. Thus, therapies directed at inhibiting TLR4 could be an alternative method to alleviate DR. How can these findings be used to improve the current DR treatment? Will such therapies be bone marrow directed? To further elucidate the function of TLR4 during DR, we performed bone marrow transplantation to create reciprocal chimeric TLR4 KO and TLR4 WT mice and then established the diabetic retina mouse model using STZ treatment. The findings will deepen the knowledge about the mechanism of DR and also provide novel strategies to treat DR.

## 2. Materials and Methods

### 2.1. Mice and Reagents

Ten-week-old male C3H/HeN mice (Animal Center affiliated with Wuhan University) and C3H/HeJ mice (Animal Center of the Military Medicine Science Institute) with bodyweights between 18 and 25 grams were housed in an SPF environment under a 12 hr light and 12 hr dark cycle. All animal protocols were approved by the Animal Care and Use Committee of the Huazhong University of Science and Technology (HUST). The ^60^Co*γ* radiation therapy instrument was provided by the cancer center of Wuhan Union Hospital. The STZ solution was purchased from Peking. The One-Touch Ultra automatic blood glucose monitoring system was obtained from Johnson Medical Instrument Limited (China). TNF-*α*, IL-1*β*, and MIP-2 ELISA kits and the ICAM-1 antibody were purchased from R&D Systems (USA), and the primers for the amplification of the* VEGF* and* HIF1*-*α* genes were purchased from Qiagen (USA). The type LZL-1 2 surgical microscope was from Zhenjiang (China), and the FEI Tecnai G12 electron microscope was from Tecnai (The Netherlands).

### 2.2. Bone Marrow Transplantation and Establishment of the Diabetes Mellitus Model

**Figure 1:**
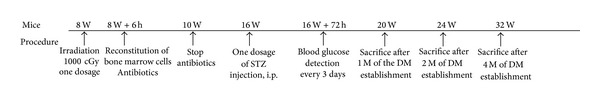
The study flow chart of the whole experiment.

Chimeric mice were produced by transferring donor bone marrow cells into irradiated recipient animals using combinations of TLR4 wild type (WT) and mutant type (Mut) mice in the following donor/recipient groups: WT/WT, WT/Mut, Mut/Mut, and Mut/WT (Figure 1). Each recipient mouse was exposed to a one-time lethal exposure of 1000 cGy ^60^Co*γ* at a dosage of 100 cGy/min for ten min at 6 h before receiving 0.5–1 × 10^6^ bone marrow cells via the tail vein. The bone marrow cells were prepared from the tibia and femur bones of the donor mice under sterile conditions. The tibias and femurs were excised and bisected under sterile conditions. The bone marrow was flushed using RPMI medium 1640 plus 5% calf serum. The marrow cells were collected, and red blood cells were lysed. Then, the cells were adjusted to a final concentration of 10^7^ cells/mL and each mice was injected with 0.1 mL. Eight weeks after the bone marrow cell transfer, peripheral blood samples were obtained to verify the success of the engraftment. Then, these chimeric mice were injected with STZ (200 mg/kg, i.p., one dose) to induce DM. Blood glucose levels were monitored 72 hrs after the injection. Mice with blood glucose concentrations lower than 16.7 mmol/L received repeated doses of STZ (70 mg/kg, i.p.) until their blood glucose levels reached 16.7 mmol/L. Insulin was not administered to the mice during the course of the experiment to mimic a long natural history of DM and its associated microvascular complications. Samples were collected at 1, 2, and 4 months after the successful establishment of the DM model. Mice were divided into 4 DM groups: WT/WT (donor/recipient), WT/Mut, Mut/Mut, and Mut/WT. Each group contained 18 mice, and each time point had 6 mice from each group. In total, 72 chimeric mice were used (shown in Figure 1).

### 2.3. The Cytokine Levels in the Retina

Supernatants from homogenized retinal tissues were collected to determine the TNF-*α*, IL-1*β*, and MIP-2 levels. ELISA assays were conducted according to the manufacturer's instructions and measured at 450 nm in a Bio-Rad ELISA reader. The results were analyzed using Bio-Linx Software (Bio-Rad, USA).

### 2.4. Retinal Expression of VEGF, HIF1-*α* mRNA

Total RNA was isolated from 0.1 mg of tissue using Trizol reagent (Gibco) following the manufacturer's protocol. One-step reverse transcription polymerase chain reaction (RT-PCR) with real-time detection was performed using the QuantiTect SYBR Green RT-PCR Kit (Qiagen) on the BIO-RAD CFX96 real-time amplification system (USA) according to the manufacturer's instructions. Cytokine expression was detected using a commercial Quantitec Primer Assay (Qiagen).

### 2.5. Electron Microscope Imaging of the Retina

After anesthetization using pentobarbital, the eyes were harvested, placed on ice, and fixed with Karnovsky's solution for transmission electron microscope observation. The tissues were cut into small pieces, stored in 2% glutaraldehyde, fixed with osmium tetroxide, and embedded in an Epon 812 mixture. Samples were sectioned using an ultramicrotome, stained with uranyl acetate and lead citrate, and examined under an FEI Tecnai G2 electron microscope (FEI, Eindhoven, The Netherlands).

### 2.6. Statistics

The data are expressed as the means ± SE. Differences between any two groups were determined by the* t*-test. Differences among multiple groups were determined by one-way ANOVA test. *P* < 0.05 was considered statistically significant (SPSS10.0 software, Public Hygiene and Health Academy in Tongji Medical College Affiliated with Huazhong University of Science and Technology).

## 3. Results and Discussion

### 3.1. Blood Glucose Levels and Mortality after the Induction of Diabetes Mellitus

After bone marrow transplantation, all mice were housed under SPF conditions in the Animal Center of Tongji Medical College, HUST. All mice survived for 8 weeks. Then, the murine DM model was successfully induced by STZ injection. The blood glucose levels fluctuated between 16.6 mmol/L and 33.1 mmol/L, with an average level of 24.8 mmol/L. One month after the STZ injection, the mortality of the DM mice was 0 and increased to 4.17% at the end of 2 months [1 mouse in the Mut/Mut group died at 22 weeks (1/24)] after injection. The mortality reached 29.17% after 4 months [1 mouse in the WT/WT group died at 31 weeks and 2 mice in each of the other 3 groups died during the last week (7/24)] without the subcutaneous injection of insulin to control the blood glucose levels. After the STZ injection, the mice exhibited typical DM symptoms, such as polyuria, polydipsia, polyphagia, and marasmus. No difference in the blood glucose level was found among the groups of mice.

### 3.2. TLR4 in Bone Marrow-Derived Cells Plays a Key Role in the Angiogenesis of the Retina, as Visualized by Transmission Electron Microscopy

**Figure 2:**
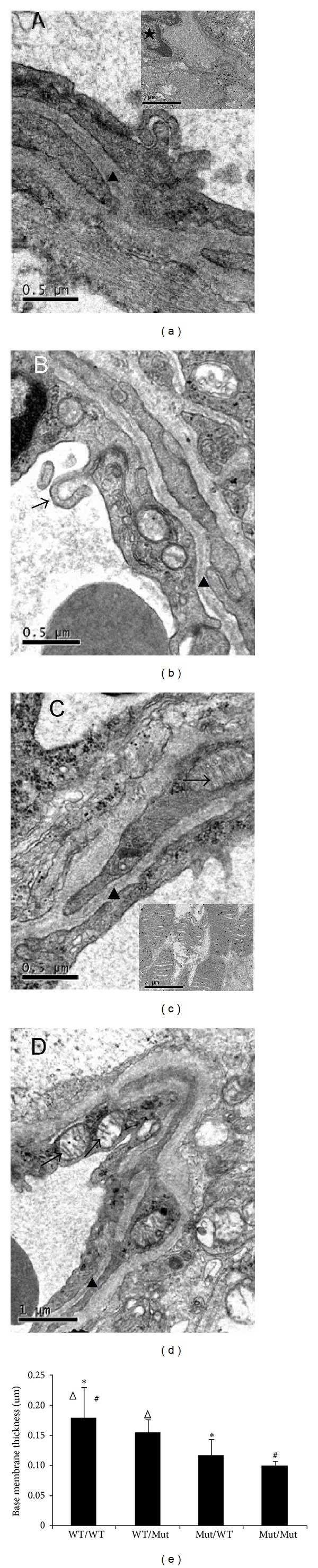
Ultrastructural signs of the blood retina barrier. (a to d) Representative electron micrographs of the retinal capillary ultrastructure from the inner layer are shown. (a) Capillary from the WT/WT (donor/recipient) group, showing a thick BM (0.18 *μ*m, arrowhead) with pericyte edema (∗); inset, monocyte infiltration in the retinal tissue (★). (b) Capillary from the WT/Mut group. Protrusion of some villi into the lumen in endothelial cells (arrow). Reduced thickening of the basal membrane (0.15 *μ*m, arrowhead). (c) Capillary from the Mut/WT group, showing a relatively thin BM (0.12 *μ*m, arrowhead). Part of the crest of the mitochondria is shortened (arrow); inset, the obvious swelling of the mitochondrial crest. (d) Capillary from the Mut/Mut group, showing a thin BM (0.10 *μ*m, arrowhead). The structure of the mitochondria is intact (arrow, magnification is indicated by the bar in the figure, *n* = 3 mice/group). (e) Vascular basement membrane thickness was measured in 12 retinal vessels from each group (from three mice). Basement membrane thickness was measured at 5 locations around the perimeter of the vessel and averaged to obtain a value for each vessel. *P < 0.01, WT/WT group compared with the Mut/WT group, ^#^ *P* < 0.01, WT/WT group compared with the Mut/Mut group, and ^Δ^ *P* < 0.01, WT/WT group compared with the WT/Mut group.

Morphological changes in the precapillary arterioles, capillaries, and venules with diameters less than 100 *μ*m were detected before injuries to the visual field or fundus became visible by ophthalmoscopic examination or fluorescein angiography. Using transmission electron microscopy, we examined the retinal ultrastructure of the mice at the end of 4 months after the STZ injection. In the WT/WT mice, thickening of the basal membrane of small vessels with or without narrowing or closure of the lumen was observed (Figure 2(a)). The nuclear membrane structure was not integrated, and the intranuclear gap was wider. In some fields, massive swelling in the mitochondria or pericytes was observed (Figure 2(a) inset). Multifoci-responsive microvilli pointing to the lumen were observed in the endothelial cells, which is rather specific to DR. Compared with the WT/WT mice, the Mut/Mut mice showed much milder changes (Figure 2(d)). Reconstruction of the irradiated WT mice with TLR4 mutant bone marrow cells (the Mut/WT group) significantly reversed the pathological changes observed in the WT/WT mice (Figure 2(b)). These results strongly suggest that TLR4 in bone marrow-derived cells indeed contributes to the progression of DR.

### 3.3. Transplantation of TLR4 WT Bone Marrow-Derived Cells into Mutant Mice Restores Cytokine Release into the Retina

**Figure 3:**
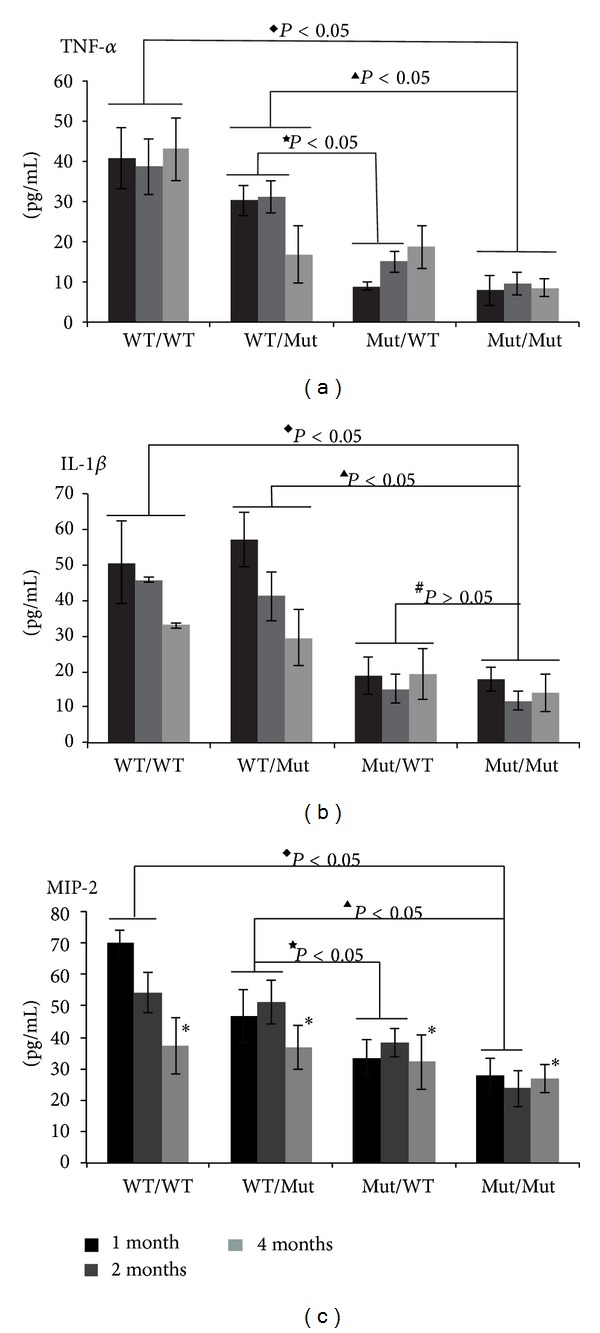
Levels of the inflammatory mediators TNF-*α*, IL-1*β*, and MIP-2 in whole retinas from each group as detected by ELISA. The data are represented as the means ± SE (4–6 mice per group). ^◆^ *P* < 0.05, compared between WT/WT and Mut/Mut groups in the same time point; ^▲^ *P* < 0.05, compared between WT/Mut and Mut/Mut groups in the same time point; ^★^ *P* < 0.05, compared between WT/Mut and Mut/WT groups in the same time point; ^#^ *P* > 0.05, compared between Mut/WT and Mut/Mut groups in the same time point; *P > 0.05, ANOVA, compared among the four groups.

The underlying mechanism of the DR pathogenesis still remains unknown given that DR results from multifactorial and complicated crosstalk among many factors. Even as early as 2001, the pathological basis of DR was noted to be a type of subacute or chronic inflammatory response [16]. To investigate the retinal inflammatory state, we measured the expression of some typical inflammatory mediators: TNF-*α*, IL-1*β*, and MIP-2. As shown in Figure 3, the levels of these inflammatory mediators were much higher in the WT/WT mice than in the Mut/Mut mice (^◆^ *P* < 0.05), which is somewhat consistent with a previous study [17]. However, after engraftment of the irradiated TLR4 mutant mice with bone marrow-derived TLR4 WT cells in the WT/Mut group, we restored the released cytokines in these mutant mice almost to the levels observed in the WT/WT mice (^▲^ *P* < 0.05). However, the cytokine release decreased dramatically when the irradiated WT mice received grafts with the bone marrow cells from TLR4 mutant mice in the Mut/WT group (^★^ *P* < 0.05, ^#^ *P* < 0.05).

Early changes in the retinal MIP-2 levels are similar to those of TNF-*α* and IL-1*β*. However, during the later stages (4 months after the induction of DM), no difference in the MIP-2 levels was observed among the 4 groups (*P > 0.05, ANOVA). One possible explanation is that MIP-2 exerts its function mainly in the early stage to recruit inflammatory cells to the highly inflamed retina (Figure 1(a) inset). During the later stage, MIP-2 function may be partially replaced by TNF-*α* because this cytokine also has chemotactic effects and could drive DR progression. Throughout DR progression, the TNF-*α* levels remained very high. These results indicated that bone marrow-derived TLR4 controls the initiation and progression of DR. This result agrees with other studies demonstrating that TLR4 in bone marrow-derived cells plays vital roles in the release of cytokines under many sterile inflammatory conditions [18–22].

However, other reports indicate that TLR4 in both bone marrow-derived and parenchymal cells could be involved in such inflammatory conditions. Delineating the roles of TLR4 from different cell origins is very complicated [22, 23].

### 3.4. Transplantation of TLR4 WT Bone Marrow-Derived Cells into Mutant Mice Restored the Expression of Genes Related to the Angiogenesis of Small Vessels

**Figure 4:**
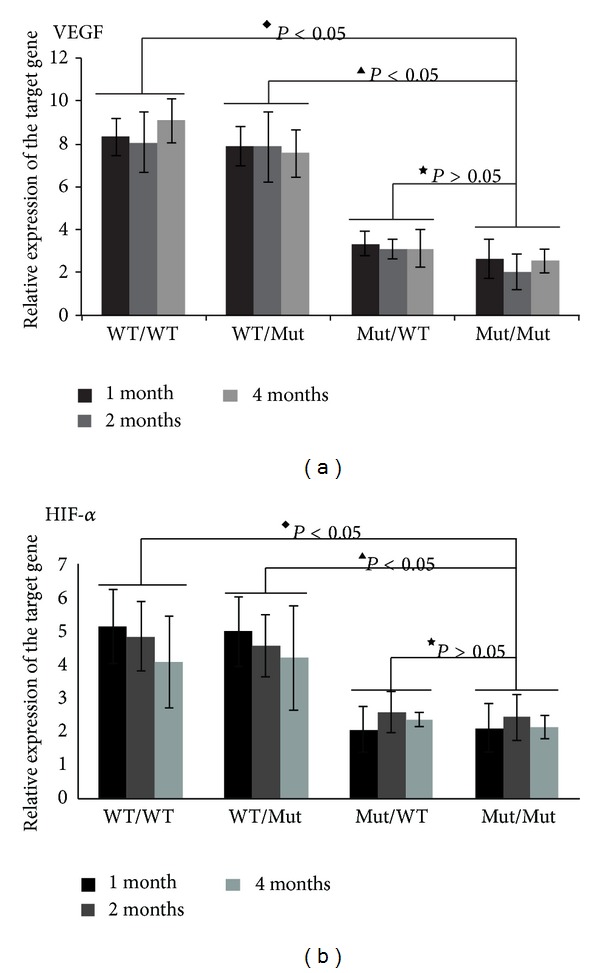
VEGF and HIF-1*α* mRNA expression in whole retinal tissue as detected by qt-PCR. The data are represented as the means ± SD (4–6 mice in each group), ^◆^ *P* < 0.05, compared between WT/WT and Mut/Mut groups; ^▲^ *P* < 0.05, compared between WT/Mut and Mut/Mut groups; ^★^ *P* > 0.05, compared between Mut/WT and Mut/Mut groups in the same time point.

Vascular endothelial growth factor (VEGF) is the most potent factor for stimulating physiological and pathological angiogenesis. The key pathological change, namely, retinal neovascularization, is associated with DM progression and is regulated by VEGF and hypoxia inducible factor-1*α* (HIF-1*α*) [24, 25]. Because the angiogenesis of small vessels is widely associated with the progression of DM, the ongoing retinopathy, and the obvious deoxygenation due to the malformation of the new vasculature, we chose to measure the VEGF and HIF-1*α* levels in the retinal tissue [26]. Because the VEGF and HIF-1*α* levels in a single mouse retina are too low to be detected by western blot or ELISA (data not shown), we used real-time quantitative PCR to detect the changes in the VEGF and HIF-1*α* mRNA levels. Our results indicate that the expression of both genes is much higher in the WT/WT mice than in the Mut/Mut group (Figure 4: ^◆^ *P* < 0.05). Grafting bone marrow-derived cells from WT mice into irradiated TLR4 mutant mice in the WT/Mut group restored the expression of these two genes (^▲^ *P* < 0.05). Grafting bone marrow-derived cells from TLR4 mutant mice into irradiated TLR4 WT mice in the Mut/WT group failed to increase the expression of these two genes (^★^ *P* > 0.05). These results suggest that TLR4 in bone marrow-derived cells is crucial for retinal neovascularization during DR. However, DM alone is known to hinder the mobilization of cells from the bone marrow as monocytes or PMN [27]. How should this issue be clearly addressed? There are still many questions to be answered especially under some persistent inflammatory state as DR.

In summary, the present study demonstrated for the first time that TLR4 in bone marrow-derived cells plays essential roles in the progression of DR and can be targeted for the prevention or treatment of DR. However, the present study focused only on TLR4 in bone marrow-derived cells during DR pathogenesis [28]. We cannot exclude the contribution of TLR4 in other cell types, such as retinal pigment epithelial cells, mononuclear cells, and gliocytes, to the development of DR [29]. Some of these cell types could also elicit very powerful immune responses, such as cytokine release [30]. Even in the retina, two distinct populations of myeloid-derived cells exist: perivascular cells (PVCs) and microglia (MG) [31]. The crosstalk between TLR4 in these cells and other immune effectors/immunomodulators remains unconfirmed and needs to be further explored.

## Abbreviations

DR: Diabetic retinopathy
TLR4: Toll-like receptor 4
TLRs: Toll-like receptors
KO: Knockout
WT: Wide type
TNF-*α*: Tumor necrosis factor-alpha
IL-1*β*: Interleukin-1 beta
MIP-2: Macrophage inflammatory protein 2
VEGF: Vascular endothelial growth factor
HIF-1*α*: Hypoxia inducible factor-1 alpha
STZ: Streptozotocin.
